# Medical Practice by Companies

**Published:** 1899-04-08

**Authors:** 


### MEDICAL PRACTICE BY COMPANIES.

The Lord Chancellor's Bill to prohibit the profession of a physician, surgeon, dentist, or midwife being carried on by a company has been issued. The following is the operative clause: "It shall be unlawful for a company under the Companies' Acts, 1862 to 1898, to carry on the profession or business of a physician, surgeon, dentist, or midwife, and if any company contravenes this enactment it shall be liable on summary conviction to a fine not exceeding £5 for every day during which the contravention happens." This ought to put a stop to what threatens to be a very serious evil.

